# A Novel LAMP-Based Lateral Flow Dipstick Technique for Rapid and Visual Detection of *Burkholderia gladioli* pv. *cocovenenans*

**DOI:** 10.3390/molecules31152664

**Published:** 2026-07-30

**Authors:** Baoqing Zhou, Xiang Nie, Hongwei Fan, Xudong Mao, Zhongxu Zhan, Cheng Zhou, Jingwen Xiong, Jiatian Gao, Mengxiang Zhang, Li Li, Bei Gan, Xin Wu

**Affiliations:** 1 Office of Science and Technology, Jiangxi General Institute of Testing and Certification, Nanchang 330052, China; bqnxzhou@163.com (B.Z.); 18727629229@163.com (X.N.); hovyfan@163.com (H.F.); maoxudong_ustb@163.com (X.M.); 17779801366@163.com (Z.Z.); z_cancan1018@163.com (C.Z.); 15079138095@163.com (J.X.); 2 School of Biology and Biological Engineering, South China University of Technology, Guangzhou 510006, China; 3 National Quality Inspection and Testing Center for Fruit and Vegetable Products and Processed Foods, Jiangxi General Institute of Testing and Certification, Nanchang 330052, China; 4 Nanchang Key Laboratory of Food Rapid Testing, Jiangxi General Institute of Testing and Certification, Nanchang 330052, China; 5 State Key Laboratory of Food Science and Resources, Nanchang University, Nanchang 330047, China; gaojiatian1104@163.com (J.G.); zmengx202407@163.com (M.Z.); 15083515471@163.com (L.L.)

**Keywords:** *Burkholderia gladioli* pv. *cocovenenans*, novel molecular target, LAMP, lateral flow dipstick, visual method

## Abstract

*Burkholderia gladioli* pv. *cocovenenans* (*B. gladioli* pv. *cocovenenans*), the most representative pathovar of *B. gladioli*, is capable of producing thermostable and highly virulent bongkrekic acid, which can result in multiple organ failure and an extremely high case fatality rate in humans. This research aims to mine specific targets for *B. gladioli* pv. *cocovenenans* and develop a rapid and accurate assay for the detection of *B. gladioli* pv. *cocovenenans* in food. Two novel specific target genes (*group_0918* and *group_0110*) were successfully screened through pan-genome analysis, and the excellent specificity and sensitivity of both targets were verified in comparison to PCR assays. Based on these targets, a loop-mediated isothermal amplification (LAMP) method was developed to specifically identify *B. gladioli* pv. *cocovenenans*. In pure culture and spiked fresh black fungus samples, the LAMP assay achieved a limit of detection (LOD) of 4.1 × 10^1^ CFU/mL and 4.1 × 10^2^ CFU/g for the detection of *B. gladioli* pv. *cocovenenans*, respectively. To enable point of care testing, a LAMP-based lateral flow dipstick(LFD) assay was established with a detection sensitivity of 4.1 × 10^3^ CFU/g in fresh black fungus. Tests on naturally infected food samples demonstrated that the developed LAMP and LAMP-assisted visual detection assays can serve as an effective alternative for the accurate identification of *B. gladioli* pv. *cocovenenans*. The novel molecular detection technology established in this study offers an efficient and reliable detection method for the prevention and control of *B. gladioli* pv. *cocovenenans* contamination in food, which holds significant application value and promotion prospects.

## 1. Introduction

*Burkholderia gladioli* (*B. gladioli*) is a Gram-negative bacterium widely found in air, soil, and aquatic environments [1,2]. According to host specificity, this species is divided into four distinct pathovars: *B. gladioli* pv. *alliicola*, *B. gladioli* pv. *agaricicola*, *B. gladioli* pv. *Cocovenenans*, and *B. gladioli* pv. *gladioli* [3,4]. Among them, *B. gladioli* pv. *cocovenenans*, formerly classified as *Pseudomonas cocovenenans* subsp. *farinofermentans* in China, is a highly virulent pathovar that causes severe foodborne infections and intoxications in humans [5]. The mortality rate of this pathogen surpasses 40%, which is significantly higher than that of prevalent food-borne pathogens such as *Listeria monocytogenes*, *Salmonella*, and *Staphylococcus aureus* [6,7]. *B. gladioli* pv. *cocovenenans* secretes two harmful toxins, bongkrekic acid (BA) and toxoflavin (TF), which target key human organs such as the liver, brain, and kidneys [8]. Individuals infected with this pathovar typically present with hepatic coma and neurological dysfunction, with eventual death caused by respiratory failure [9,10]. Foodborne poisoning caused by *B. gladioli* pv. *cocovenenans* has long been endemic in Indonesia and Asia [11]. In 2015, a severe food poisoning outbreak induced by this pathogen occurred in Mozambique, Africa, causing 75 deaths [12]. In 2024, the first fatal case of bongkrekic acid poisoning was reported in North America [13]. Statistics from the national public health emergency surveillance system indicated that a total of 30 foodborne poisoning incidents triggered by *B. gladioli* pv. *cocovenenans* occurred in China during 2005–2020, causing 188 illnesses and 85 deaths [14]. Foodborne diseases attributed to this pathovar are commonly identified in black fungus, *Tremella fuciformis*, fermented rice and flour products, and various starchy foods [9]. Hence, rapid, accurate, and sensitive identification of this pathovar has become an urgent demand, which plays a key role in mitigating relevant food safety hazards and reducing the risk of food poisoning.

Currently, traditional culture methods serve as the gold standard for the detection of *B. gladioli* pv. *cocovenenans*. As stipulated in the Chinese national standard GB 4789.29-2020 [15], the detection procedure, which involves pre-enrichment culture, bacterial isolation and purification, biochemical identification, and toxigenic analysis, is cumbersome to operate and time-consuming. Matrix-Assisted Laser Desorption/Ionization Time-of-Flight Mass Spectrometry (MALDI-TOF MS) technology is available for the rapid identification of *B. gladioli* pv. *cocovenenans* [16]. Nevertheless, this approach depends on specialized apparatus and costly databases. Moreover, pre-enrichment culturing must be carried out before detection to obtain purified single colonies. With the development of molecular biology, molecular detection methods represented by polymerase chain reaction (PCR) [17], loop-mediated isothermal amplification (LAMP) [18], and rolling circle amplification (RCA) [19] have gradually become mainstream due to their advantages of low cost, rapidity, and sensitivity. However, traditional PCR methods rely on professional thermal cyclers, making them unable to meet the needs of primary-level testing. LAMP is a nucleic acid amplification method characterized by low reliance on professional equipment and high sensitivity. It can achieve 10^9^–10^10^ fold amplification under isothermal conditions in a short time [20]. The traditional LAMP method typically involves direct analysis of amplification products via gel electrophoresis, which can easily lead to aerosol contamination in the experimental environment [21]. To mitigate the risk of cross-contamination during experiments, Kim et al. (2023) [22] developed a fluorescence visualization method based on LAMP technology for the rapid detection of *Salmonella* Thompson. By incorporating a fluorescent probe, this approach significantly reduces the probability of cross-contamination in the analysis process; furthermore, it achieves a limit of detection (LOD) of 3 CFU/mL without the need for enrichment treatment. Zheng et al. (2025) [23]. designed biotin and FITC-labeled primers and successfully established a LAMP lateral flow dipstick (LFD) platform for rapid, sensitive detection of *B. gladioli* pv. *cocovenenans*. The detection sensitivity reached 2.5 pg/μL for purified pathogen DNA and 6.68 × 10^2^ CFU/25 g for artificially contaminated rice noodle samples, which is 10 times more sensitive than conventional PCR. Yao et al. (2025) [24] developed a dual-readout lateral flow biosensor for *B. gladioli* pv. *cocovenenans* detection based on the combined colorimetric and magnetic properties of polydopamine-coated Fe_3_O_4_ magnetic nanospheres (Fe_3_O_4_@PDA MNSs). The method allowed for a quantitative LOD of 2.32 × 10^3^ CFU/mL and a visual LOD of 1.0 × 10^4^ CFU/mL. Compared with the gold-nanoparticle-based lateral flow dipstick (AuNPs-based LFD) platform, this method achieved an approximately ninefold increase in detection sensitivity. Therefore, molecular detection methods based on LAMP and LFD platforms have demonstrated significant application potential in the rapid and accurate identification of pathogenic bacteria.

**Figure 1:**
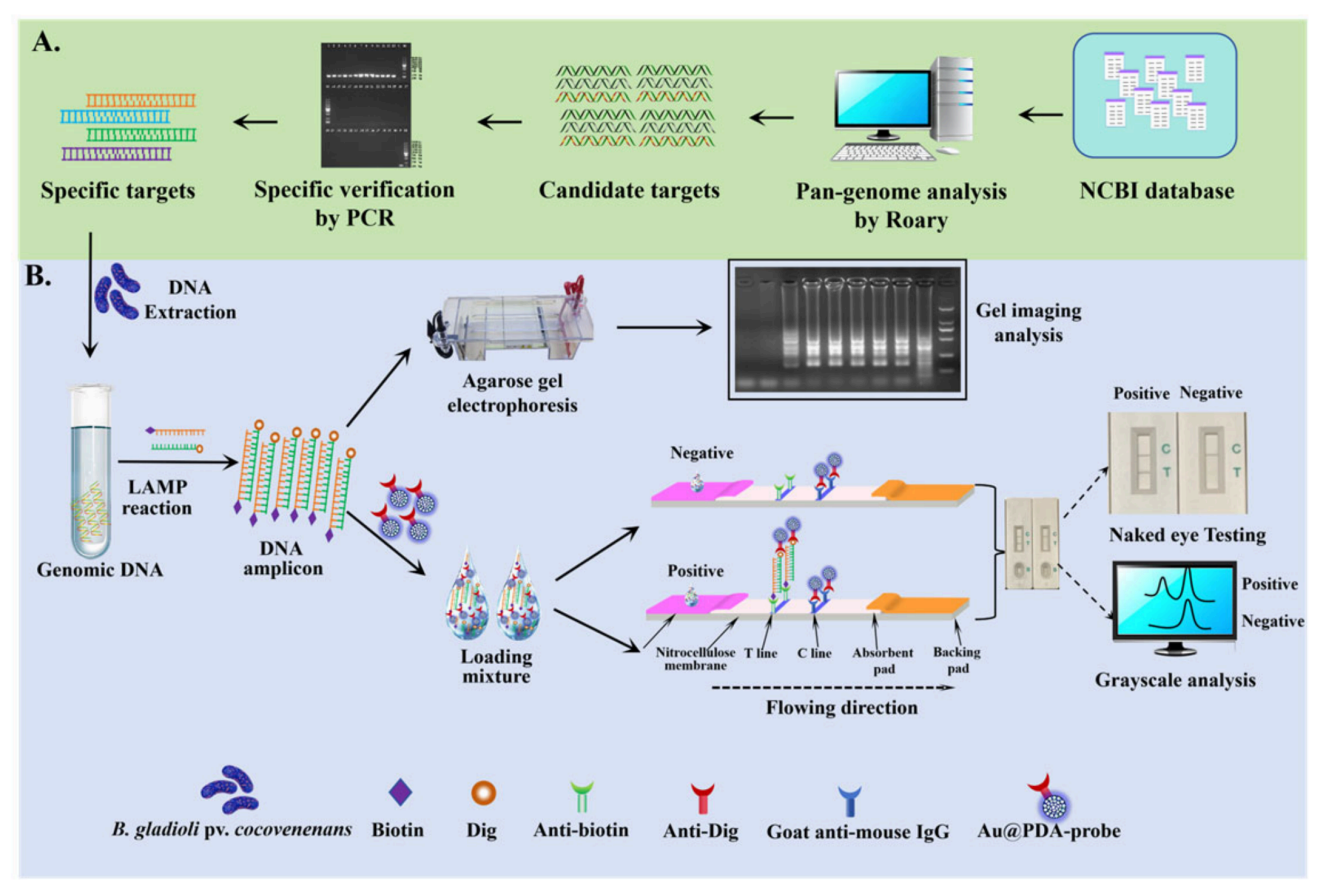
Schematic illustration of experimental workflow. (**A**) The workflow of special target screening for *B. gladioli* pv. *cocovenenans*; (**B**) establishment of a molecular method based on LAMP and lateral flow dipstick.

Specific molecular targets are critical for guaranteeing the specificity and sensitivity of molecular detection assays. Molecular targets and supporting primers for one-step PCR of *B. gladioli* pv. *cocovenenans* remain limited, with routine markers confined to *16S rRNA*, *23S rRNA,* and the *16S-23S rRNA* spacer region [25,26]. These conserved sequences share high homology across pathovar subtypes and other *Burkholderia* species, thereby hindering accurate interspecific discrimination. A qPCR method was developed, targeting the *bonM* gene within the bongkrekic acid biosynthetic gene cluster, for the rapid screening of *B. gladioli* pv. *cocovenenans* in food samples [27]. Nevertheless, as a virulence gene, *bonM* harbors widespread sequence variation and substantial genetic divergence. Over-reliance on this single target impedes accurate identification and fails to meet practical detection requirements. Driven by the rapid development of high-throughput sequencing and bioinformatics, pangenomic analysis techniques with superior capabilities in global genetic profiling have been extensively utilized for dissecting bacterial genetic variation, gene function enrichment, and species evolutionary traceability [28]. Our previous research successfully mined specific molecular targets for *Staphylococcus* spp. [29], *Salmonella* spp. [30], and *Listeria* spp. [31] based on pan-genome analyses, enabling accurate and rapid identification of target bacteria in complex food matrices. Therefore, based on the public genomic data from the National Center for Biotechnology Information (NCBI, https://www.ncbi.nlm.nih.gov/) and our group’s previously obtained whole-genome sequencing data of *B. gladioli* pv. *cocovenenans*, pan-genome analysis can be employed to precisely mine specific molecular detection targets. We will further develop user-friendly, rapid, cost-effective, and highly sensitive LAMP assays and LFD colorimetric methods that will meet the practical demands for the accurate identification and quantitative detection of *B. gladioli* pv. *cocovenenans* (Figure 1).

## 2. Results

### 2.1. Screening of Novel Specific Targets for B. gladioli pv. cocovenenans

As listed in Table S1, 501 genome sequences were collected to screen novel specific target genes, consisting of 489 from *Burkholderia* strains and 12 from other common pathogenic bacteria. According to the selection criteria, two of the evaluated genes were successfully identified as candidate targets (Table S2). Functional prediction showed that both *group_0918* and *group_0110* encode hypothetical proteins, and identifying their specific coding regions may facilitate gene structure–function analyses to elucidate the unique metabolic characteristics of *B. gladioli* pv. *cocovenenans* [32]. A local BLASTN program was used to verify the specificity of these genes for the detection of *B. gladioli* pv. *cocovenenans*. As shown in Table S3, the two candidate targets displayed 100% detection rate in *B. gladioli* pv. *cocovenenans* strains and 100% specificity against non-target strains. By comparison, the reported target *BonM* showed 100% presence in target strains but only 98.3% specificity in non-target strains in local BLASTN analysis [27]. Overall, our candidate targets exhibited superior performance in both specificity and positive coverage relative to the previously reported marker.

To select an optimal specific target for the detection of *B. gladioli* pv. *cocovenenans*, the primer’s specificity and sensitivity for each target were determined. As illustrated in Table 1 and Figure S1, both primer pairs successfully amplified the target amplicon across all tested strains of *B. gladioli* pv. *cocovenenans*, while no amplification products were observed in all non-target strains, indicating high primer specificity. The qPCR detection method based on two molecular targets exhibited a limit of detection (LOD) of 4.1 × 10^2^ CFU/g for *B. gladioli* pv. *cocovenenans* in artificially contaminated samples (Figure S2). Based on amplification efficiency and LAMP primer design performance, *group_0918* was selected for subsequent reactions.

### 2.2. Verification of LAMP-Based Visual Assay

To achieve novel LAMP-based visual detection of *B. gladioli* pv. *cocovenenans*, LAMP primers were designed based on the specific molecular target *group_0918* (Table 2), and Figure S3 illustrates the main steps of LAMP and the positioning of the LAMP-modified primers on the target DNA sequence.

The optimal LAMP reaction conditions were determined as follows: The total reaction volume of 25 μL contained 2.5 μL of 10× isothermal amplification buffer, 1 μL of Bst 2.0 WarmStart DNA polymerase (8000 U/mL), primer mixtures of F3/B3:LB/LF:FIP/BIP = 1:4:8 (1 = 0.2 µmol/L), 1 μL of Mg^2+^ (125 mmol/L), 5 μL of dNTPs (5 mmol/L), 1 μL of target DNA, and ultrapure water to complete the final volume. The LAMP optimal reaction was performed at 65 °C (Figure S4), followed by inactivation at 85 °C for 10 min. Subsequently, 80 μL of 10-fold-diluted LAMP products was thoroughly mixed with 2 μL of anti-digoxigenin (DIG) antibody-modified Au@PDA probes for subsequent LFD visual detection. In the presence of target DNA, LAMP amplicons dual-labeled at both ends with digoxin (DIG) and biotin were generated, which specifically bound to Au@PDA probes in the reaction system. During lateral flow, the biotin-labeled amplicons were specifically captured by immobilized anti-biotin antibodies on the T line, while free Au@PDA probes were recognized and bound by goat anti-mouse secondary antibodies on the C line, thereby forming two distinct visible bands. In contrast, no LAMP was triggered without target DNA. Only free Au@PDA probes were captured by antibodies on the C line, yielding a single distinct control line band. To prevent the non-specific adsorption of large LAMP amplicons onto the membrane matrix and to ensure capillary flow efficiency, we optimized the running buffer with surfactants.

### 2.3. Sensitivity of LAMP-Based Visual Method

**Figure 2:**
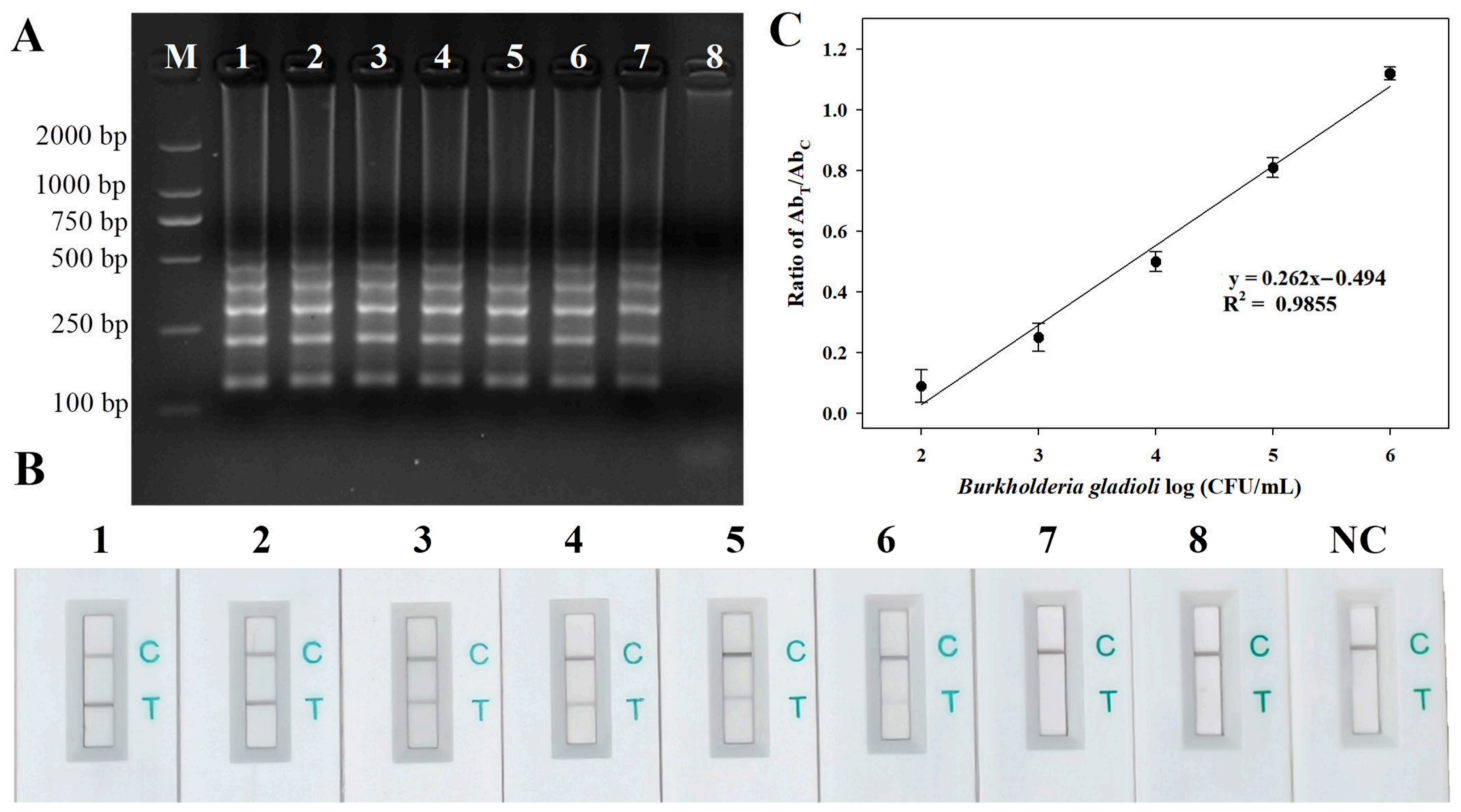
Sensitivity evaluation of conventional LAMP and LAMP-based visual assay in pure culture. (**A**) The results of the conventional LAMP assay, lanes 1–8: genomic DNA of target bacteria from 10^7^ to 10^0^ CFU/mL, lanes M: DL2000 DNA marker. (**B**) The results of the LAMP-based visual assay, No. 1–8: genomic DNA of target bacteria from 10^7^ to 10^0^ CFU/mL, NC: negative control. (**C**) Establishment of standard curves using the ratio values of AB_T_/AB_C_ in relation to the logarithmic numbers of *Burkholderia gladioli* pv. *cocovenenans* cells within a range of 10^2^~10^6^ CFU/mL; AB_T_: the absorbance intensity of the T line; AB_C_: the absorbance intensity of the C line. All results were collected in triplicate, with error bars representing the standard deviation of three independent replicates.

The sensitivity of LAMP-based visual assay was determined using concentration gradients ranging from 10^0^ to 10^7^ of *B. gladioli* pv. *cocovenenans*. The plate counting result on LB agar indicated that the concentration of fresh bacterial liquid of *B. gladioli* pv. *cocovenenans* was 4.1 × 10^8^ CFU/mL. All genomic DNA was extracted for traditional LAMP and LAMP visual analysis. As shown in Figure 2A, typical ladder-like bands were observed in lanes 1 to 7, while no specific amplicons were detected in lane 8. The LOD of the conventional LAMP assay was calculated to be 4.1 × 10^1^ CFU/mL for pure culture samples. For the LFD method, two bands were observed at the concentration of 4.1 × 10^2^ CFU/mL, while only a single quality control band was present in the 4.1 × 10^1^ CFU/mL group and the blank control. And the absorbance ratio of the T line (AB_T_) to the C line (AB_c_) at the concentration of 10^2^ CFU/mL was significantly higher than those obtained at lower concentrations of 10^2^ CFU/mL and the blank control group. Therefore, the LOD of this LFD method was determined to be 4.1 × 10^2^ CFU/mL, with a linear range covering 4.1 × 10^2^–4.1 × 10^6^ CFU/mL in pure culture samples (Figure 2B,C).

**Figure 3:**
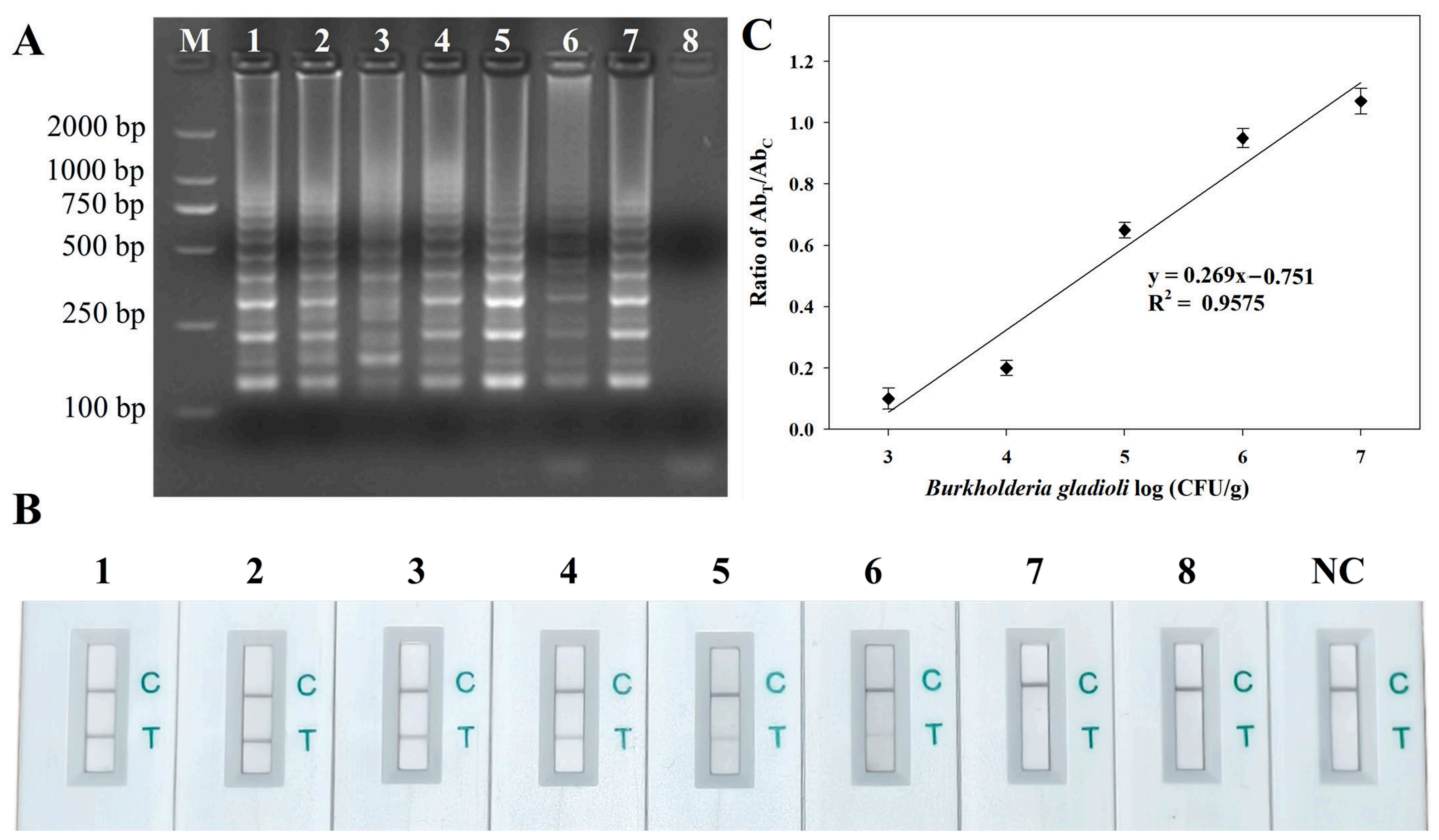
Sensitivity evaluation of conventional LAMP and LAMP-based visual assay in artificially spiked black fungus. (**A**) The results of the conventional LAMP assay, lanes 1–8: genomic DNA of target bacteria from 10^8^ to 10^1^ CFU/g, lanes M: DL2000 DNA marker. (**B**) The results of the LAMP-based visual assay, No. 1–8: genomic DNA of target bacteria from 10^8^ to 10^1^ CFU/g; NC: negative control. (**C**) Establishment of standard curves using the ratio values of AB_T_/AB_C_ in relation to the logarithmic numbers of *Burkholderia gladioli* pv. *cocovenenans* cells within a range of 10^3^~10^7^ CFU/g; AB_T_: the absorbance intensity of the T line; AB_C_: the absorbance intensity of the C line. All results were collected in triplicate, with error bars representing the standard deviation of three independent replicates.

For LOD evaluation in artificially spiked samples, fresh spiked black fungus samples with concentration gradients of 10^1^ to 10^8^ CFU/g were obtained. In Figure 3A, the LOD of the conventional LAMP assay was calculated to be 4.1 × 10^2^ CFU/g in a spiked black fungus sample (Figure 3A). The LOD of the LFD method was determined to be 4.1 × 10^3^ CFU/g, with a linear range covering 4.1 × 10^3^–4.1 × 10^7^ CFU/g (Figure 3B,C) and a coefficient of determination (R^2^) of 0.95 (Figure 3C). It should be noted that although the LFD method exhibited a linear detection range of 4.1 × 10^3^–4.1 × 10^7^ CFU/g with an R^2^ of 0.95 (Figure 3C), residual analysis revealed an uneven distribution along the concentration gradient, with substantially larger absolute residuals at low bacterial loads, indicating poor linearity within this low-concentration interval. Specifically, the nonlinear deviation was most pronounced at concentrations below 10^4^ CFU/g, whereas acceptable linearity was observed across the higher concentration range (10^5^–10^7^ CFU/g). This nonlinear deviation was likely attributable to the complex food matrix effect: DNA extraction by the boiling lysis approach failed to sufficiently remove endogenous inhibitory components, including polysaccharides and proteins, and these residual impurities suppress LAMP more severely when template DNA is limited, thus accounting for the compromised linearity at low concentrations. Despite this limitation in quantitative linearity at low concentrations, the LFD biosensor achieved an LOD comparable to that of the conventional LAMP method (only one order of magnitude higher). The modest loss of analytical sensitivity is an inherent feature of lateral flow platforms, primarily resulting from partial signal attenuation during chromatographic migration and specific capture of amplicons on the test strip. Nevertheless, the instrument-free format and simple operational workflow of this LFD-based detection system still satisfy the requirements of POCT. The application of commercial DNA extraction kits could further improve genomic DNA recovery and reduce co-extracted inhibitors, thereby alleviating matrix suppression and enhancing linear fitting performance in complex sample testing in future applications.

### 2.4. Specificity of LAMP-Based Visual Method

**Figure 4:**
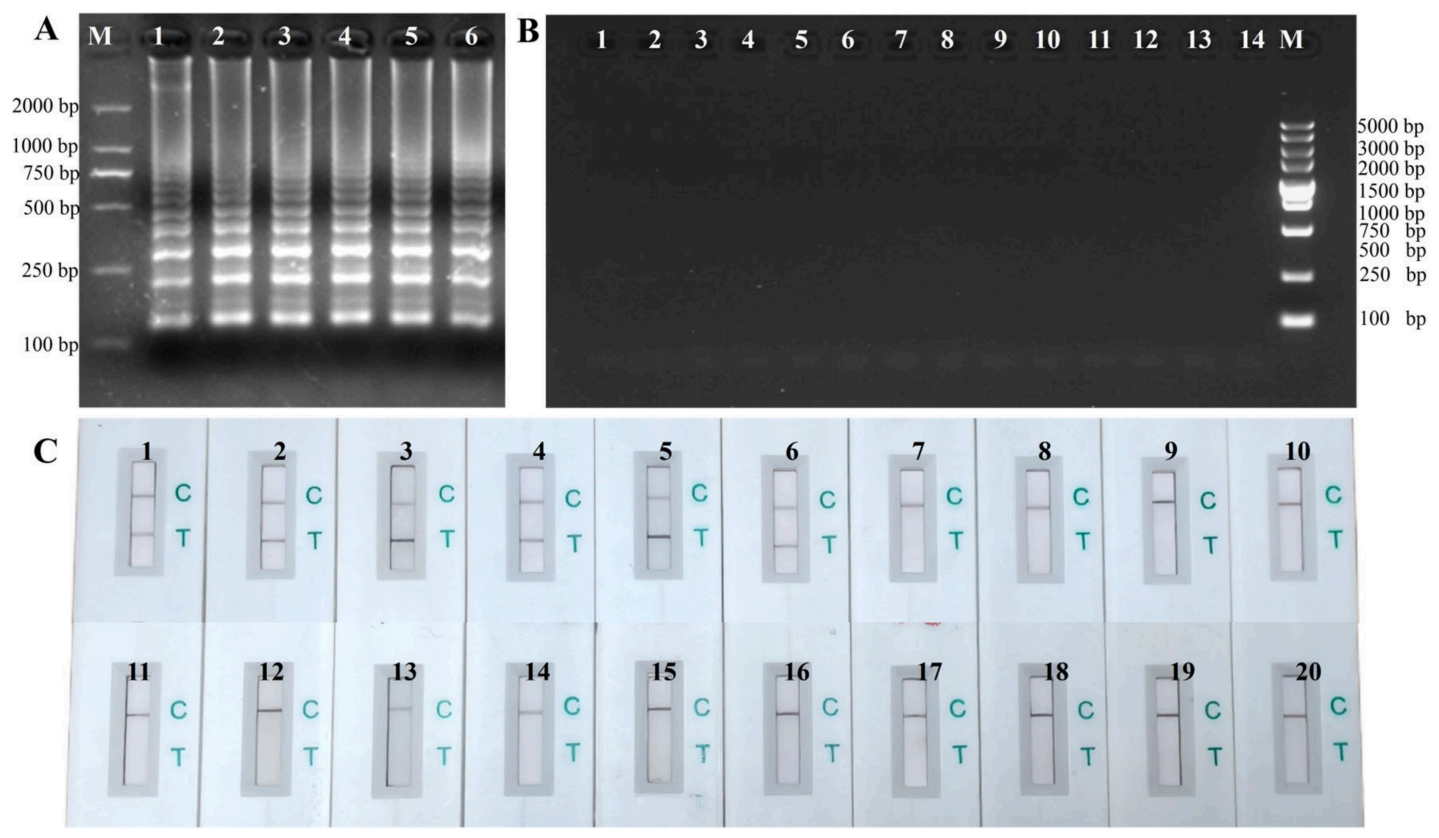
Specificity validation of conventional LAMP and LAMP-based visual assay. (**A**) The specificity results of the conventional LAMP assay, lanes 1–6: target *B. gladioli* strains, lanes M: DL2000 DNA marker. (**B**) Lanes 1–14: non-target strains, lanes M: DL5000 DNA marker. (**C**) The specificity results of the LAMP-based visual assay: No. 1–6: target *B. gladioli* strains; No. 7–20: non-target strains.

To determine the specificity of the LAMP visual assay, genomic DNA from six target *B. gladioli* pv. *cocovenenans* strains and 14 non-target *B. gladioli* pv. *cocovenenans* strains was extracted (Table S4). As shown in Figure 4, positive results were obtained for all target *B. gladioli* pv. *cocovenenans* strains, while negative signals were obtained for non-target bacterial strains, indicating excellent specificity for the identification of *B. gladioli* pv. *cocovenenans*.

### 2.5. Application of the LAMP Visual Method in Natural Samples

**Figure 5:**
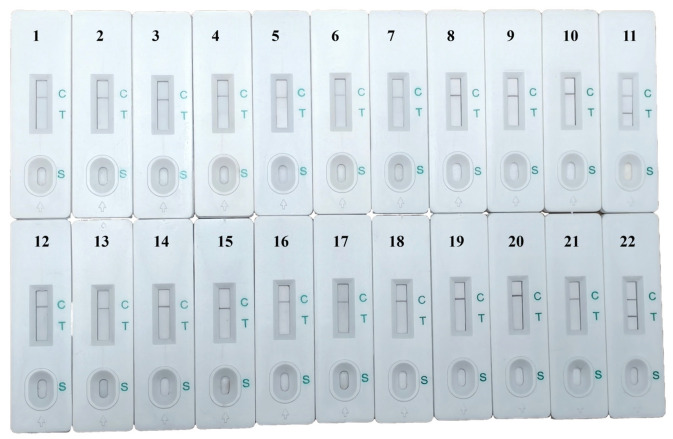
Natural sample analysis. No 1–10: unknown fresh black fungus; No 11: spiked fresh black fungus; No 12–21: unknown wet rice noodles; No 22: spiked wet rice noodles.

To assess the application of LAMP visual detection, a total of 22 samples were prepared for DNA extraction. All results are shown in Figure 5; only two positive control samples exhibited positive test results for *B. gladioli* pv. *cocovenenans*, whereas the remaining 20 food samples produced negative results using this established assay. Although the sensitivity, specificity, and efficiency were all 100% in the 22 tested samples (Table 3), the small number of positive samples (*n* = 2) resulted in a relatively wide 95% confidence interval for sensitivity (34.2–100%), while the specificity (83.9–100%) and efficiency (85.1–100%) showed narrower intervals, reflecting the limited sample size and the high consistency with the standard method.

## 3. Discussion

*B. gladioli* pv. *cocovenenans* mainly contaminates coconuts, edible fungi, cereal crops, and tuber plants, and it readily colonizes humid, organic-rich habitats and synthesizes lethal toxins, thereby resulting in severe economic losses and serious threats to public health [33,34]. At present, the gold standard for the detection of *B. gladioli* pv. *cocovenenans* involves a series of procedures, including pre-enrichment, biochemical identification, serotyping, and liquid chromatography analysis. These conventional approaches are time-consuming, laborious, and costly, and they fail to meet the demand for large-scale sample screening. PCR-based molecular detection provides an alternative strategy for the identification of *B. gladioli* pv. *cocovenenans* [29]. Previous studies have demonstrated that genes sequencing, such as *recA*, *hisA*, *gyrB*, and 16S *rRNA,* can be used to differentiate *B. gladioli* strains [16]. Nevertheless, phylogenetic analysis based on these single gene demonstrates a complex evolutionary relationship within the genus *Burkholderia*, resulting in inaccurate strain identification. Moreover, reported targets for the specific identification of *B. gladioli* pv. *cocovenenans*, such as the *16S-23S rRNA* internal transcribed spacer (ITS) [26] and virulence gene *BonM* [27], were mined based on small-scale genomic datasets, gene fragments, or single virulence genes. High sequence homology of these targets among closely related *Burkholderia* species, coupled with frequent genetic variations during natural evolution, substantially reduces their specificity and accuracy in practical detection [35]. Therefore, mining of novel conserved molecular targets is essential to establish a reliable approach for accurate detection of *B. gladioli* pv. *cocovenenans*.

In contrast to conventional strategies that rely on coding sequences or individual virulence genes, this study employed pan-genome analysis for target mining based on whole-genome sequence alignment. This robust approach has been widely adopted to identify molecular markers associated with bacterial virulence, serotyping, molecular subtyping, and antibiotic resistance [32]. In this study, a total of 501 genomic sequences, including 489 from *Burkholderia* strains and 12 from other common pathogenic bacteria, were retrieved from the NCBI database and in-house sequencing data. Two novel, specific molecular targets were identified in this study. These targets predominantly derive from genes acquired via interspecific horizontal gene transfer and exhibit stable conservation in strains displaying the associated phenotypic traits [36]. The newly identified targets provide an efficient and convenient alternative for the accurate detection of *B. gladioli* pv. *cocovenenans*. It is noteworthy that both target genes characterized in this study correspond to hypothetical proteins of unknown function. Characterization of these protein-coding regions is crucial for unraveling their gene architecture and biological functions, which will significantly enhance our comprehension of the metabolic and phylogenetic features of *B. gladioli* pv. *cocovenenans* [37].

With the continuous expansion of genomic databases, some previously reported species-specific nucleotide targets have been confirmed to exhibit cross-reactivity with non-target genomes [16]. It is essential to combine bioinformatics analysis and large-scale strain PCR validation to evaluate the specificity and sensitivity of molecular targets. As shown in Table S3, alignment against the local genomic database revealed that the specific targets identified in our study achieved 100% positive coverage and specificity, whereas previously reported targets displayed slight sequence overlap with other species [27]. PCR validation conducted with 13 target strains and 27 non-target strains indicated that the novel targets achieved positive amplification across all target strains and demonstrated high specificity towards non-target isolates. To achieve rapid and accurate identification of *B. gladioli* pv. *cocovenenans*, a qPCR system was established based on the newly screened specific targets. In artificially contaminated fresh black fungus samples, the LOD of the qPCR assay was 4.1 × 10^2^ CFU/g for *B. gladioli* pv. *cocovenenans*. Compared with previously reported methods, the established qPCR assay presents a simpler workflow while maintaining high accuracy [38]. To further reduce reliance on sophisticated instruments, a LAMP method was developed using these molecular targets. Under optimal reaction conditions, the detection sensitivities for pure cultures and artificially contaminated fresh black fungus samples were 3.8 × 10^1^ CFU/mL and 3.8 × 10^2^ CFU/g, respectively. The specificity results exhibited no cross-reactivity toward related strains, verifying the favorable stability and reliability of this assay. However, conventional molecular detection techniques rely on agarose gel electrophoresis and fluorescent dye analysis, which fail to meet the requirements of on-site rapid detection. Furthermore, aerosol contamination is easily generated during nucleic acid amplification, leading to false-positive results [21].

To realize low-cost point-of-care testing (POCT), we designed matched linker probes and colorimetric probes and constructed a LAMP-based visualized nucleic acid strip platform (market survey: ¥132 for culture method, ¥50 for commercial qPCR assay, ¥20 for this LAMP-strip assay per test). Sensitivity evaluation showed that the LAMP visualized assay achieved LODs of 3.8 × 10^2^ CFU/mL and 3.8 × 10^3^ CFU/g in pure bacterial cultures and artificially contaminated black fungus samples, respectively. The assay displayed excellent specificity against 14 non-target strains. Its analytical performance, including specificity, LOD, and field applicability, was systematically compared with previously reported LAMP methods from Zheng et al. (2025) and Yao et al. (2025), with full details provided in Table S5 [23,24]. The testing results of 22 natural food samples demonstrated that the established visual LAMP method exhibited high consistency with the standard culture assay, suggesting promising prospects for practical applications. In this study, the performance verification of the LAMP assay was conducted at a pilot scale, with only 22 samples from two food matrices (fresh black fungus and wet rice noodles) included. The validation results cannot fully represent the assay performance across all food matrices potentially contaminated by *B. gladioli* pv. *cocovenenans*. Subsequent studies will expand the sample size and cover more food categories to further confirm the universality and stability of the established method.

Undeniably, the detection method established in this study still involves pre-enrichment and nucleic acid amplification procedures, which impose certain limitations on detection efficiency. Existing studies have integrated the CRISPR/Cas system with microfluidic chips for *B. gladioli* pv. *cocovenenans* detection. This approach enables discrimination of multiple subtypes of *B. gladioli* pv. *cocovenenans* without pre-amplification, and it remarkably improves detection efficiency and practical applicability [39,40]. In subsequent research, we will employ innovative specific molecular targets, integrate the CRISPR/Cas system with microfluidic chips and intelligent analytical technologies, and develop a high-throughput detection system for *B. gladioli* pv. *cocovenenans* to promote the prevention and control of food safety risks.

## 4. Materials and Methods

### 4.1. Mining of Novel Specific Targets for B. gladioli pv. cocovenenans

To identify specific targets of *B. gladioli* pv. *cocovenenans*, a total of 501 genome sequences were retrieved for pan-genome analysis (Table S1). The dataset comprises 489 taxonomically validated *Burkholderia* genomes (all publicly available strains from the NCBI database plus our in-house sequencing data, collected exhaustively to capture intra-genus genetic diversity) and 12 common foodborne pathogenic strains serving as negative controls for specificity validation. The pan-genome analysis based on a local script written in the Perl programming language was used to achieve rapid and effective screening [32]. Briefly, all genome sequences were annotated to establish an analysis library using the Prokka v1.11, an open-source prokaryotic genome annotation tool developed by Torsten Seemann (Monash University, Australia). Then, Roary v3.11.2, an open-source prokaryotic pan-genome analysis tool primarily developed by Andrew J. Page (The Wellcome Trust Sanger Institute, Hinxton, UK), was used to perform gene sequence alignment with a BLASTP identity cut-off of 85% [36]. The obtained gene coverage data were used to analyze the presence or absence of genes. Based on the principle of 100% gene coverage in the respective target strains and 0% in the nontarget strains, candidate targets of *B. gladioli* pv. *cocovenenans* were initially identified. An in-house whole-genome database of foodborne pathogenic bacteria (https://www.mbiosh.com/data-analysis/database/, accessed on 18 July 2025) and the NCBI online BLASTN program (against the non-redundant nucleotide (nt) database) were used to further validate the specificity of the target genes.

### 4.2. Bacterial Strains and Genomic DNA Extraction

The strains used in this study are listed in Table 1, including 13 target *B. gladioli* strains and 27 non-target strains. All strains were stored in 30% glycerin broth (*v*/*v*) at −80 °C before use. All test bacteria were grown in Luria–Bertani (LB) medium (Guangdong Huankai Co., Ltd., Guangzhou, China) at 37 °C with 180 rpm/min. Bacterial genomic DNA was extracted from each strain using the conventional boiling lysis method and stored at −20 °C prior to use [41].

### 4.3. Specificity and Sensitivity Verification of Candidate Targets

To evaluate the specificity of targets, the genomic DNA of 40 strains of experimental bacteria was extracted as templates for ordinary PCR reactions (Table 1). The reactions contained 5 µL of 2× Premix Taq (Takara Biomedical Technology Co., Ltd., Beijing, China), 0.5 µL of each forward and reverse primer (10 μM), 1 µL of each genomic DNA, and 3 µL of ultrapure water to a final volume of 10 µL. A T100™ Thermal Cycler (Bio-Rad Laboratories, Hercules, CA, USA) was used to perform PCR amplification as follows: predenaturation for 3 min at 95 °C; 35 cycles of denaturation for 30 s at 95 °C; annealing for 30 s at 58 °C; and extension for 1 min at 72 °C; and a final extension at 72 °C for 5 min. Agarose gel electrophoresis (2.5%) and the Gel Doc™ XR+ Gel Documentation System (Bio-Rad Laboratories, Hercules, CA, USA) were used to analyze the PCR products. The criteria for great specificity targets were as follows: A single target band was amplified from the DNA of the target bacteria, while no amplification bands were observed from the DNA of non-target bacteria [42]. To evaluate the sensitivity of the molecular targets, a qPCR assay was established using the designed specific primer pairs. A serial 10-fold dilution of *B. gladioli* pv. *cocovenenans* from artificially contaminated samples, with concentrations ranging from 10^8^ CFU/mL to 10^2^ CFU/mL, was prepared for qPCR analysis. The total 20 μL reaction system contained 10 μL of TB Green™ Premix Ex Taq™ II (TaKaRa Biotech, Dalian, China), 0.5 μM of each primer, and 1 μL of template DNA, with ultrapure water added to make up the final volume. Amplification was carried out on a LightCycler^®^ 96 real-time PCR instrument (Roche, Basel, Switzerland) with the following thermal profile: initial denaturation at 95 °C for 3 min, followed by 40 cycles of 95 °C for 10 s and 60 °C for 45 s. The acquired fluorescence data were analyzed using LightCycler^®^ 96 Software v1.1.0.1320 (Roche, Basel, Switzerland). All experiments were performed in three replicates, with genomic DNA of the *B. gladioli* pv. *cocovenenans* standard strain as the positive control and nuclease-free water as the no-template negative control per amplification run.

### 4.4. Development of a LAMP Assay for B. gladioli pv. cocovenenans Visualization Detection

#### 4.4.1. LAMP System

The LAMP primer pairs for *B. gladioli* pv. *cocovenenans* specific targets were designed through online software (http://primerexplorer.jp/e/, accessed on 25 August 2025) and synthesized by Sangon Biotech Co. Ltd., Shanghai, China (Table 2). The LAMP reaction system was prepared with minor modifications according to a previous study [43]. A temperature gradient ranging from 58 °C to 68 °C was set to screen the optimal amplification condition of the LAMP assay, and the temperature producing the clearest and brightest ladder-like bands was determined as the optimal reaction temperature. The total LAMP reaction volume of 25 µL contained a 10× isothermal amplification buffer, MgSO_4_ (Sangon Biotech Co., Ltd., Shanghai, China), dNTPs (5 mmol/L, Sangon Biotech Co., Ltd., Shanghai, China), Bst 2.0 WarmStart DNA polymerase (New England Biolabs, Ipswich, MA, USA), outer primers F3/B3, inner primers FIP/BIP, loop primers LB/LF, template DNA (genomic DNA from 10^5^ CFU/mL target strains), and ultrapure water. All amplification reactions were set with genomic DNA of the *B. gladioli* pv. *cocovenenans* standard strain as the positive control and nuclease-free water as the no-template negative control. The reactions were amplified at a constant temperature of 58–68 °C and inactivated at 85 °C for 10 min. All amplified products (template: genomic DNA from 10^5^ CFU/mL target strains) were identified via 2% agarose gel electrophoresis.

To mitigate aerosol contamination and prevent false positives in the LAMP system, reagent preparation, sample processing, and amplification/detection were performed in physically separated rooms with unidirectional workflow. During the preparation of the reaction system, aerosol-resistant filter tips were used exclusively, and each reaction mixture was overlaid with mineral oil to prevent contamination. Workbenches and pipettes were regularly treated with DNA decontamination solution before and after experiments.

#### 4.4.2. Development of a LAMP-Based Visual Method

As shown in Figure 1, the LFD platform was independently developed in our laboratory, consisting of three core components: a sample pad, an absorbent pad, and a nitrocellulose (NC) membrane. With the use of an XYZ3060 dispensing system (BioDot, Irvine, CA, USA), 0.8 mg/mL of anti-biotin antibodies and 1.2 mg/mL of goat anti-mouse secondary antibodies were separately coated on the NC membrane to prepare the test (T) line and control (C) line, with the dispensing rate set at 30 μL/s. The treated NC membrane was dried at 37 °C for 12 h. All test strips were hermetically preserved in a desiccator before use.

For visual detection, the LAMP products were diluted 10-fold with sterile water after amplification. Briefly, 80 μL of the diluted solution was thoroughly mixed with 2 μL of 1 mg/mL anti-digoxigenin (DIG) antibody-modified Au@PDA probes. The mixture was loaded onto the pre-prepared sample pad, incubated at room temperature for 15 min, and bands of the T and C lines were judged by the naked eye. For quantitative detection, a strip reader (FIC-S1, Fenghang Technology Co., Ltd., Hangzhou, China) was used to detect and analyze the absorbance intensity. The ratio of the AB_T_ to AB_C_ was utilized to eliminate background interference. All samples were tested in triplicate under consistent experimental conditions (including standardized LAMP product dilution, fixed probe dosage, consistent loading volume, and 15 min room-temperature incubation) to guarantee result repeatability.

### 4.5. Sensitivity Validation of the LAMP Visual Method

To determine the pure culture sensitivity of the LAMP assay, 10-fold serial dilutions of *B. gladioli* suspensions ranging from 10^0^ to 10^8^ CFU/mL were prepared. All genomic DNA was extracted for the traditional LAMP assay and LAMP visual analysis.

For LOD evaluation in artificially spiked samples, a total of 25 g of black fungus determined to be negative for *B. gladioli* by standard culture methods was homogenized in 225 mL of sterile saline solution. Fresh cultures of *B. gladioli* CICC25108 suspensions were mixed with the homogenate to prepare varied substrate concentrations of 10^0^~10^7^ CFU/g, and all genomic DNA was extracted for the traditional LAMP assay and LAMP visual analysis.

### 4.6. Specificity Validation of the LAMP Visual Method

The specificity of the developed assay was determined using 6 target *B. gladioli* strains and 14 non-target strains (Table S4). All genomic DNA for the traditional LAMP assay and LAMP visual analysis was extracted using the conventional boiling lysis method.

### 4.7. Natural Sample Analysis

For natural sample visual detection, a total of 22 food samples, including 11 fresh black fungus (10 unknown real samples and 1 positive control sample) and 11 wet rice noodles (10 unknown real samples and 1 positive control sample), were purchased from a local agricultural market (Nanchang, China). Subsequently, 25 g of each sample was added to sterile Erlenmeyer flasks containing 225 mL of GVC enrichment broth, thoroughly mixed, and cultured at 37 °C for 4–6 h. Each 1 mL of resuspension containing *B. gladioli* pv. *cocovenenans* was collected for DNA extraction and then for LAMP visual analysis. For comparison, the above 22 samples were detected using the Chinese national standard method (GB 4789.29-2020) [15].

## 5. Conclusions

In summary, specific molecular targets for *B. gladioli* pv. *cocovenenans* were successfully mined based on pan-genome analysis, and a novel LAMP-visual detection method was established for the on-site detection of *B. gladioli* pv. *cocovenenans* in food. Compared with the traditional culture method, the developed molecular assay is cost-effective and easy to operate, enabling specific and accurate identification of *B. gladioli* pv. *cocovenenans* in samples. In artificially contaminated fresh black fungus samples, the LAMP assay exhibited an LOD of 4.1 × 10^2^ CFU/g for *B. gladioli* pv. *cocovenenans*, while the visual detection combined with the LFD platform achieved a sensitivity of 4.1 × 10^3^ CFU/g. This developed LAMP visual strategy presents a credible alternative to the conventional culture method, and it provides valuable technical support for exploring the virulence profiles and epidemiological distribution of *B. gladioli* pv. *cocovenenans* in food matrices.

## Figures and Tables

**Table 1:** Strain information for the specific target analysis of *B. gladioli* pv. *cocovenenans* in this study.

Organism	No.	Bacterial Species	Strain	Source	Special Target for PCR	
					*Group_0918*	*Group_0110*
*Burkholderia* spp.	1–4	*Burkholderia gladioli* pv. *cocovenenans*	CICC25108, CICC25126, CICC25131, CICC25131	a	+	+
	5–13	*Burkholderia gladioli* pv. *cocovenenans*	BGC_0110, BGC_0111, BGC_0112, BGC_0113, BGC_0114, BGC_0115, BGC_0116, BGC_0117, BGC_0118	b	+	+
	14	*Burkholderia gladioli*	BG_0119	b	−	−
	15	*Burkholderia cepacia*	CICC10857	a	−	−
	16	*Burkholderia contaminans*	CICC23882	a	−	−
	17	*Burkholderia aenigmatica*	CICC24958	a	−	−
	18	*Burkholderia multivorans*	CICC25193	a	−	−
	19	*Burkholderia metalliresistens*	CICC10561	a	−	−
	20	*Burkholderia cenocepacia*	CICC25294	a	−	−
	21	*Burkholderia cenocepacia*	CICC25262	a	−	−
Non-target strains	22	*Pseudomonas aeruginosa*	ATCC 10104	c	−	−
	23	*Pseudomonas aeruginosa*	ATCC 15442	c	−	−
	24	*Escherichia coli*	CMCC 44103	d	−	−
	25	*Cronobacter sakazakii*	ATCC 29544	c	−	−
	26	*Bacillus cereus*	ATCC 14579	c	−	−
	27	*Campylobacter jejuni*	ATCC 6633	c	−	−
	28	*Vibrio parahemolyticus*	ATCC 33847	c	−	−
	29	*Listeria monocytogenes*	ATCC 19114	c	−	−
	30	*Listeria monocytogenes*	ATCC 19113	c	−	−
	31	*Staphylococcus aureus*	ATCC 29213	c	−	−
	32	*Staphylococcus epidermidis*	SE-1	b	−	−
	33	*Staphylococcus* *haemolyticus*	SH-1	b	−	−
	34	*Proteus mirabilis*	CMCC 49005	d	−	−
	35	*Salmonella enteritidis*	CMCC 50335	d	−	−
	36	*Proteus vulgaris*	CMCC 49027	d	−	−
	37	*Flavobacterium columnare*	ATCC 23463	c	−	−
	38	*Flavobacterium aquatile*	ATCC 11947	c	−	−
	39	*Flavobacteriumjohnsoniae*	ATCC 17061	c	−	−
	40	*Flavobacterium hydatis*	ATCC 29551	c	−	−

a: CICC, China Center of Industrial Culture Collection, China; b: laboratory isolate; c: ATCC, American Type Culture Collection, USA; d: CMCC, China Medical Culture Collection, China; +/−: positive and negative signals.

**Table 2:** Sequences involved in this study.

Name	Sequence (5′-3′)
*G0918*-PCR-122F	AAACCACCCGCAACCTGAT
*G0918*-PCR-122R	GAAACGCAACACCTCCTCCA
*G0110*-PCR-258F	GACCGCCTTCTTCTCCCTGC
*G0110*-PCR-258R	TGGATGATCGCGTGATTCGT
*G0918*-LAMP-F3	CTGGCCGCGTATTTCCG
*G0918*-LAMP-B3	CGCAGCAGCGTATAGACAC
*G0918*-LAMP-FIP	Biotin-CTCGTCGTCGTCGGCGATGT-GAAGACGACGGCGAGGAGA
*G0918*-LAMP-BIP	Dig-ATGAGCGAGGAGGAATTGCTCG-CAGGTTGCGGGTGGTTTC
*G0918*-LAMP-LB	GCAGCAGGTTCAGCAGGT
*G0918*-LAMP-LF	GCAATGCGTGATGCTGTTGTT

**Table 3:** Statistical analysis between conventional LAMP, LAMP visual assay, and the standard method in actual sample detection.

Sample Types	Sample Name	Number of Samples	Standard Method		Conventional LAMP Assay		LAMP Visual Assay		Sensitivity (%) (95% CI)	Specificity (%) (95% CI)	Efficiency (%) (95% CI)
			+	−	+	−	+	−			
Natural negative samples (total *n* = 20)	Fresh black fungus	10	0	10	0	10	0	10	100 (34.24–100)	100 (83.89–100)	100 (85.14–100)
	Wet rice noodles	10	0	10	0	10	0	10			
Spiked samples (total *n* = 2)	Spiked fresh black fungus	1	1	0	1	0	1	0			
	Spiked wet rice noodles	1	1	0	1	0	1	0			

Note: Values in parentheses represent 95% Wilson score confidence intervals for sensitivity and specificity.

## Data Availability

The original contributions presented in this study are included in the article/Supplementary Materials. Further inquiries can be directed to the corresponding authors.

## Supplementary Materials

The following supporting information can be downloaded at https://www.mdpi.com/article/10.3390/molecules31152664/s1.
