# Supplementary material for: A Novel LAMP-Based Lateral Flow Dipstick Technique for Rapid and Visual Detection of Burkholderia gladioli pv. cocovenenans
Source: Molecules. 2026 Jul 30;31(15):2664. doi: 10.3390/molecules31152664 (PMC13467799; doi:10.3390/molecules31152664)
Supplement: Supplementary file 1 [file molecules-31-02664-s001.zip › molecules-4388494-supplementary.pdf]

Supplementary materials

A Novel LAMP-Based Lateral Flow Dipstick Technique for Rapid and Visual Detection of *Burkholderia gladioli* pv. *cocovenenans*

Baoqing Zhou [1,2,3,4,†], Xiang Nie [1,3,4,†], Hongwei Fan [1,3,4], Xudong Mao [1], Zhongxu Zhan [1,3,4], Cheng Zhou [1,3,4], Jingwen Xiong [1,3,4], Jiatian Gao [5], Mengxiang Zhang [5], Li Li [5], Bei Gan [1,3,4,*] and Xin Wu [1,3,4,*]

[1] Office of Science and Technology, Jiangxi General Institute of Testing and Certification, Nanchang 330052, China; bqnxzhou@163.com (B.Z.); 18727629229@163.com (X.N.); hovyfan@163.com (H.F.); maoxudong_ustb@163.com (X.M.); 17779801366@163.com (Z.Z.); z_cancan1018@163.com (C.Z.); 15079138095@163.com (J.X.)

[2] School of Biology and Biological Engineering, South China University of Technology, Guangzhou 510006, China

[3] National Quality Inspection and Testing Center for Fruit and Vegetable Products and Processed Foods, Jiangxi General Institute of Testing and Certification, Nanchang 330052, China

[4] Nanchang Key Laboratory of Food Rapid Testing, Jiangxi General Institute of Testing and Certification, Nanchang 330052, China

[5] State Key Laboratory of Food Science and Resources, Nanchang University, Nanchang 330047, China; gaojiatian1104@163.com (J.G.); zmengx202407@163.com (M.Z.); 15083515471@163.com (L.L.)

* Correspondence: ganbeikm@126.com (B.G.); 18770022505@163.com (X.W.)

† These authors contributed equally to this work.

Table S1. Information of *Burkholderia* spp. and non-target bacteria for specific target analysis during this study.

Organism	Species	Assembly
Burkholderia spp.	*Burkholderia gladioli* pv. *cocovenenans* (n=19)	GCA_042604585.1, GCA_048917665.1, GCA_048917655.1, GCA_048918525.1, GCA_055386035.1, GCA_003713105.1, GCA_048917685.1, GCA_000194745.1 BGC_0110, BGC_0111, BGC_0112, BGC_0113, BGC_0114, BGC_0115, BGC_0116, BGC_0117, BGC_0118, BGC_0119, BGC_0120
	Other Burkholderia gladioli pathovars (n=22)	GCA_016698705.1, GCA_000959725.1, GCA_036839165.1, GCA_036837415.1, GCA_053470145.1, GCA_053470105.1, GCA_055397955.1, GCA_026651625.1, GCA_014263355.1, GCA_052222725.1, GCA_048565175.1, GCA_025244785.1, GCA_023658595.1, GCA_050613125.1, GCA_042604595.1, GCA_054187845.1, GCA_003613595.1, GCA_002208175.1, GCA_045862465.1, GCA_002393425.1, GCA_050920555.1, GCA_029762415.1
	Burkholderia aenigmatica (n=2)	GCF_021899435.1, GCF_902833005.1
	Burkholderia ambifaria (n=9)	GCF_000019925.1, GCF_000203915.1, GCF_000959545.1, GCF_016127755.1, GCF_016458785.1, GCF_020843905.1, GCF_020843925.1, GCF_020843985.1, GCF_026958255.1
	Burkholderia anthina (n=3)	GCF_016458805.1, GCF_017514945.1, GCF_030374675.1
	Burkholderia arboris (n=3)	GCF_024381035.1,GCF_902830425.1
	Burkholderia catarinensis (n=1)	GCF_001883705.2
	Burkholderia cenocepacia (n=28)	GCA_001718895.1, GCA_001999785.1, GCA_001606135.1, GCA_001999825.1, GCA_039728355.1, GCA_021535065.1, GCA_018223805.1, GCA_025643575.1, GCA_014357995.1, GCA_018228625.1, GCA_018223765.1, GCA_041205585.2, GCA_023374855.1, GCA_023374835.1, GCA_023374335.1, GCA_001606115.1, GCA_002007585.1, GCA_001484665.1, GCA_003966315.1, GCA_001999885.1, GCA_014211915.1, GCA_003076415.1, GCA_000203955.1, GCA_000009485.1, GCA_000236215.4, GCA_001718515.1, GCA_000755725.1, GCA_000764955.1
	Burkholderia cepacia (n=30)	GCA_009586235.1, GCA_001411495.1, GCA_006094315.1, GCA_003546465.1, GCA_023374475.1, GCA_021344035.2, GCA_001718395.1, GCA_023053515.1, GCA_023374675.1, GCA_023374715.1, GCA_021484945.1, GCA_028462305.2, GCA_050632355.1, GCA_046773525.1, GCA_001718835.1, GCA_014854795.1, GCA_024600575.1, GCA_002209085.2, GCA_002407525.3, GCA_050632325.1, GCA_047902435.1, GCA_033675325.1, GCA_050613135.1, GCA_041070875.1, GCA_029962485.1, GCA_000292915.1, GCA_000974835.2, GCA_000755805.1, GCA_904865725.1, GCA_000701165.2
	Burkholderia contaminans (n=23)	GCA_040215475.1, GCA_029633825.1, GCA_054955335.1, GCA_001029145.1, GCA_029633645.1, GCA_022533485.1, GCA_018223785.1, GCA_007724625.1, GCA_009755625.1, GCA_009191525.1, GCA_044057485.1, GCA_054558305.1, GCA_029590335.1, GCA_029633805.1, GCA_003704285.1, -GCA_024661975.1, GCA_025643515.1, GCA_050632315.1, GCA_050632335.1, GCA_050632305.1, GCA_050632345.1, GCA_019915365.1, GCA_037101275.1
	Burkholderia diffusa (n=4)	GCF_001718315.1, GCF_902830825.1, GCF_902830855.1, GCF_914492725.1
	Burkholderia dolosa (n=2)	GCF_000152585.1, GCF_000959505.1
	Burkholderia glumae (n=52)	GCF_000022645.2, GCF_000960995.1, GCF_009578005.1, GCF_009857095.1, GCF_009931375.1, GCF_012974245.1, GCF_013122645.1, GCF_013307145.1, GCF_013307165.1, GCF_016026055.1, GCF_016598875.1, GCF_017858835.1, GCF_024022955.1, GCF_024662395.1, GCF_024662415.1, GCF_024662435.1, GCF_024662455.1, GCF_024662475.1, GCF_024662615.1, GCF_024662735.1, GCF_024662895.1, GCF_024663015.1, GCF_024663135.1, GCF_024663255.1, GCF_024663375.1, GCF_024663615.1, GCF_024663775.1, GCF_024663895.1, GCF_024663975.1, GCF_024663995.1, GCF_024664015.1, GCF_024664035.1, GCF_024664055.1, GCF_024664075.1, GCF_024664095.1, GCF_024664115.1, GCF_024664135.1, GCF_024664155.1, GCF_024664175.1, GCF_024664195.1, GCF_024664215.1, GCF_024664235.1, GCF_024664255.1, GCF_024664275.1, GCF_024760585.1, GCF_024760605.1, GCF_024760625.1, GCF_024760645.1, GCF_026651975.1, GCF_026651995.1, GCF_026652015.1, GCF_030505635.1
	Burkholderia humptydooensis (n=7)	GCF_000170495.1, GCF_000266985.1, GCF_001462435.1, GCF_001513745.1, GCF_002888035.1, GCF_002888095.1, GCF_016027455.1
	Burkholderia guangdongensis (n=1)	GCF_013403875.1
	Burkholderia lata (n=3)	GCF_000012945.1, GCF_001718575.1, GCF_003711145.1
	Burkholderia latens (n=9)	GCF_001525985.1, GCF_001718795.1, GCF_008802135.1, GCF_017815595.1, GCF_017815615.1, GCF_019795555.1, GCF_902499045.1, GCF_902832755.1, GCF_902832795.1,
	Burkholderia malle (n=33)	GCF_000011705.1, GCF_000015465.1, GCF_000015605.1, GCF_000015625.1, GCF_000755785.1, GCF_000755845.1, GCF_000755865.1, GCF_000755885.1,

	GCF_000756025.1, GCF_000762285.1, GCF_000959165.1, GCF_000959405.1, GCF_000959465.1, GCF_000959485.1, GCF_000959625.1, GCF_001729545.1, GCF_002345985.1, GCF_002346005.1, GCF_002346025.1, GCF_002346045.1, GCF_002346065.1, GCF_002346085.1, GCF_002346105.1, GCF_002346125.1, GCF_002346145.1, GCF_002346165.1, GCF_002346185.1, GCF_002346205.1, GCF_033870355.1, GCF_033870375.1, GCF_033870395.1, GCF_033956065.1, GCF_939576165.1
Burkholderia mayonis (n=2)	GCF_001522635.2, GCF_001523745.2
Burkholderia metallica (n=10)	GCF_001718555.1, GCF_003034145.1, GCF_013336775.1, GCF_013337715.1, GCF_020419265.1, GCF_020419365.1, GCF_030482065.1, GCF_902499065.1, GCF_902832805.1, GCF_902832815.1
Burkholderia multivorans (n=51)	GCF_000010545.1, GCF_000018505.1, GCF_000756005.1, GCF_000959525.1, GCF_001718755.1, GCF_001718995.1, GCF_003019965.1, GCF_003812365.1, GCF_003812585.1, GCF_003812965.1, GCF_008693485.1, GCF_008693525.1, GCF_009730035.1, GCF_009730175.1, GCF_009730195.1, GCF_017165175.1, GCF_017165195.1, GCF_017165475.1, GCF_017165495.1, GCF_017165515.1, GCF_017165535.1, GCF_017165555.1, GCF_023373625.1, GCF_023373645.1, GCF_023373665.1, GCF_023373685.1, GCF_023374315.1, GCF_023374355.1, GCF_023374375.1, GCF_023374395.1, GCF_023374415.1, GCF_023374435.1, GCF_023374455.1, GCF_023374495.1, GCF_023374515.1, GCF_023374535.1, GCF_023374575.1, GCF_023374595.1, GCF_023374615.1, GCF_023374635.1, GCF_023374655.1, GCF_023374695.1, GCF_023374735.1, GCF_023374755.1, GCF_023374775.1, GCF_023374795.1, GCF_023374815.1, GCF_023374875.1, GCF_023374895.1, GCF_025643495.1, GCF_025643555.1,
Burkholderia oklahomensis (n=2)	GCF_000959365.1, GCF_001522135.2
Burkholderia paludis (n=3)	GCF_000732615.1, GCF_902499105.1, GCF_902859865.1
Burkholderia perseverans (n=1)	GCF_022870505.1
Burkholderia plantarii (n=3)	GCF_000835205.1, GCF_001411805.1, GCF_030644525.1
Burkholderia orbicola (n=1)	GCF_000019505.1
Burkholderia pseudomallei (n=72)	GCF_000015905.1, GCF_000015925.1, GCF_000260515.1, GCF_000294635.1, GCF_000439695.1, GCF_000494855.1, GCF_000511895.1, GCF_000511915.1, GCF_000520895.1, GCF_000521645.1, GCF_000583835.1, GCF_000755765.1, GCF_000755825.1, GCF_000755905.1, GCF_000755925.1, GCF_000755945.1, GCF_000755965.1, GCF_000756065.1, GCF_000756085.1, GCF_000756125.1, GCF_000756145.1, GCF_000756165.1, GCF_000756185.1, GCF_000757015.2, GCF_000757035.2, GCF_000764575.1, GCF_000770395.1, GCF_000770455.1, GCF_000770495.1, GCF_000770515.1, GCF_000770535.1, GCF_000770565.1, GCF_000953095.1, GCF_000954175.1, GCF_000959125.1, GCF_000959145.1, GCF_000959185.1, GCF_000959205.1, GCF_000959225.1, GCF_000959265.1, GCF_000959285.1, GCF_000959305.1, GCF_000959345.1, GCF_001277875.1, GCF_001277895.1, GCF_001277975.1, GCF_001318245.1, GCF_001695715.1, GCF_001695735.1, GCF_001885195.1, GCF_001887555.1, GCF_001887575.1, GCF_001975065.1, GCF_001975085.1, GCF_001975105.1, GCF_001975125.1, GCF_001975145.1, GCF_001980585.2, GCF_002110945.1, GCF_002110965.1, GCF_002111005.1, GCF_002111025.1, GCF_002111045.1, GCF_002111085.1, GCF_002111105.1, GCF_002111125.1, GCF_002111145.1, GCF_002111165.1, GCF_002111205.1, GCF_002111225.1, GCF_002111245.1, GCF_002111265.1
Burkholderia pseudomultivorans (n=23)	GCF_001523105.1, GCF_001523165.1, GCF_001524505.1, GCF_001525825.1, GCF_001532035.1, GCF_001532065.1, GCF_001532085.1, GCF_001533465.1, GCF_001718415.1, GCF_015354235.1, GCF_031573355.1, GCF_031573365.1, GCF_031573395.1, GCF_031573415.1, GCF_031573425.1, GCF_031573455.1, GCF_031573465.1, GCF_031573475.1, GCF_031573555.1, GCF_031630155.1, GCF_031630195.1, GCF_902499075.1, GCF_902832925.1
Burkholderia puraquae (n=2)	GCF_002099195.1, GCF_902859845.1
Burkholderia pyrrocinia (n=5)	GCF_001028665.1, GCF_003330765.1, GCF_018417535.1, GCF_022809715.1, GCF_024732225.1
Burkholderia reimsis (n=1)	GCF_003294055.1
Burkholderia savannae (n=2)	GCF_001524445.2, GCF_001524625.2
Burkholderia seminalis (n=2)	GCF_000705535.2, GCF_001718535.1
Burkholderia singularis (n=2)	GCF_001523725.1, GCF_900176645.1
Burkholderia stabilis (n=3)	GCF_001742165.1, GCF_002356275.1, GCF_900240005.1
Burkholderia stagnalis (n=3)	GCF_001718955.1, GCF_034317865.1, GCF_902833065.1
Burkholderia territorii (n=2)	GCF_001636095.1, GCF_001718335.1
Burkholderia thailandensis (n=25)	GCF_000012365.1, GCF_000385525.1, GCF_000567905.1, GCF_000567925.1, GCF_000567945.1, GCF_000706745.1, GCF_000764595.1, GCF_000765375.1, GCF_000808035.2, GCF_000959425.1, GCF_000959605.1, GCF_001718295.1, GCF_001718595.1, GCF_001718615.1, GCF_001718635.1, GCF_002386205.1, GCF_003020025.1, GCF_003020185.1, GCF_003568605.1, GCF_003614815.1, GCF_003614835.1, GCF_011578485.1, GCF_016774495.1, GCF_033675275.1, GCF_033675305.1
Burkholderia ubonensis (n=12)	GCF_000959245.1, GCF_001718355.1, GCF_001718655.1, GCF_001718695.1, GCF_001718855.1, GCF_001718975.1, GCF_002097715.1, GCF_002362295.1,

	Burkholderia vietnamiensis (n=11)	GCF_000016205.1, GCF_000959445.1, GCF_001718495.1, GCF_001718775.1, GCF_001718815.1, GCF_001718915.1, GCF_003019945.1, GCF_020099355.1, GCF_022213445.1, GCF_023373725.1, GCF_030078435.1
Other bacteria (n=12)	*Bacillus cereus*	GCA_001635915.1
	Campylobacterjejuni	GCA_003999645.1
	Cronobacter sakazakii	GCA_001277275.1
	Escherichia coli	GCA_000005845.2
	Listeria monocytogenes	GCA_000438685.2
	Salmonella enterica	GCA_000743055.1
	Staphylococcus aureus	GCA_006094915.1
	Vibrio parahaemolyticus	GCA_001558495.2
	Yersinia pestis	GCA_003798225.1
	Bacillus subtilis	GCA_003148415.1
	Pseudomonas aeruginosa	GCA_001457615.1
	Staphylococcus epidermidis	GCA_006742205.1

Table S2. The nucleotide sequences of specific target genes for *B. gladioli* pv. *cocovenenans.*

**Name**	**Encoded protein**	**Gene location***	**Nucleotide sequences**
Group_0918	hypothetical protein	3022904 ~ 3024136	ATGGATACCGCTGTCACACCTGTCGTTCCTGCCCTCTATAGCGCGGAG-<br>TTTTTCCGCAATCGCGATCCCTACCCGACCTATCAGAAGCATCTA-<br>GAAGGTCCGGTGGTGCAGTCCTCGGCGATGATGCCCAATAC-<br>CTGGCTGATGTTCCGCTACGCCGCCTGCTCGAC-<br>GCATCTGCGCGACCCGAACCTGTCGGCCGCGCGTCCGCCCAAGCTGCTGG-<br>TACCGGTGCCCGAGGCCGACCTGCCGCGTTTCGAGGATCTGGCCTCGCAC-<br>CTGCAACGCTGGCTGCTGATGCGCGACCAGCCGAGCCACAAGCCGCTGCG-<br>CAAGCTGATGAACAAGGGCTTCTCGCCCCTCACGATCCAG-<br>CAGATGCGCCCGCGCATCGAGCTGCTGGTCGAGCGCCTGGTGGACGAC-<br>CTGCGTGCGGCGCCGGAGCCCGACATCATGCGCGACTTCGCGTATCCGC-<br>TACCAGTGCGCGTGATCTGCGAATTGCTGGGCGTGCCGGATGCACTGCAG-<br>GACCGCTGCGTGGTGCTGACCGAC-<br>GACATCGCCGTGCTGCTCGCCAACCCGCACGTCACCACGGAAGCCGCG-<br>GAGTGCGCGCAGGACGCGGTACGGGAACTGGCCGCG-<br>TATTTCCGCGACATCATCCGCGATCATGAAGACGACGGCGAGGAGAAC-<br>CTGCTGAACCTGCTGCTGCACATCGCCGACGACGACGAGCAG-<br>CAGATGAGCGAGGAGGAATTGCTCGCGCAATGCGTGATGCTGTT-<br>GTTCGCTGGCCACGAAACCACCCGCAACCTGATCGGCAACGGTGTCTATAC-<br>GCTGCTGCGCCACCCGGAATCGCTCGCGGCCGTGCGCGCCGATCCG-<br>GAGCGCGTGAAATCCGCGGTGGAGGAGGTGTTGCGTTTCGAG-<br>TCGCCGCTGCAGGCCGTGCGCCGCGTGACGACGGCCGAGGTCGAG-<br>TTCGACGGCACCCGGATTCCGGCCGGCCACACGGTGATCTTCGTCAC-<br>CGCCGCGGCGAACCGGGATCCGCGCCAGTTCGAGGATCCGGAC-<br>CGCTTCGACATCGATCGCCGGCTGAATCGCCACCTCGCGTTCGGCG-<br>CAGATCACCATGTCTGCATCGGTAGCACACTGGCGCGTCTCGAAGGG-<br>CAGATCGCGATCGACGCGATCATGCGCGCGTTCCCGAATCTGCAACTTCTT-<br>GACCAGACGCCCGACTGGCGCGGCCATGCCGCGATGCGAGGCTTCCGCAC-<br>GCTGCGCGTCGGCATCTAG
Group_0110	hypothetical protein	872277 ~ 873032	ATGGAACGACCATTTTTGAAAATATCCCAAAGCAATGAC-<br>GTATTGCTTATCGGTCTCGATCGGCCGGAAAAGCG-<br>CAATGCCATGCCATGGGCCATGCTGCAC-<br>GGATTGCGCGATGTATTTACTCGCGTGCCCGACGA-<br>TATTCGGGCCGCCGTGCTCTACAGCACGCACGAGCATTTTT-<br>GCGCGGGTCTCGATTTGAGCGAGGTGCGCGGG-<br>CAATCGGTGATCGACTGTTTCCAGTTTTTCCGTGAATGGCAGGAC-<br>GTGTTCCAGCAGATCCAGTATTGCCGCGTAC-<br>CGGTGGTCGCGGCGATCTCGGGCGCGG-<br>CAATCGGTGCGGGCCTCGAAATGTCGGCGGCCTGCCACATCCGGGTGGTG-<br>GACGAGACCGCCTTCTTCTCCCTGCCCGAGGCCGCGCGCGGCCTTTAC-<br>GTGGGGCTCGGCGCCTCGGTGCGTTTCCCGCGTCTGGCCGGCAC-<br>GGCGCTGATGACCGACATGATGCTCACGGGCCGTCGGCTGCGTGCCGAG-<br>GAAAGCGTCGCGCGCGGCGTCGCCCAGTAC-<br>CTGGTGCCGGCCGGCCGGGCCGTCGACAAGGCGCTCGAACTCGCCATCGTCG<br>CCGCCTCGAATCCGCCGATGACGAATCACGCGATCATCCAC-<br>GCGCTGCCGCGCATCGTCGAGCAGGACCCGGATCAGGGCTTCTACAC-<br>CGAAGCGCTGATCGCCGCGGTGGTCCAGTCGTCGCCCGAGACGGCGGCAC-<br>GCATGAGCGCATTCCTCGACCGCAAGAAGTAA

* The reference strain is *Burkholderia gladioli* strain Co14 (CP033430.1) for *group_0918* and *group_0110*

Table S3. Profiles of newly mined or reported *B. gladioli* pv. *cocovenenans* specific targets for target and nontarget strains.

Genus	Target gene	Presence profile in		Source
		Target strain	Non-target strain	
Burkholderia spp.	*group_0918*	19 (100 %)	482 (0 %)	This study
	group_0110	19 (100 %)	482 (0 %)	This study
	bonM	19 (100 %)	482 (1.7 %)	[28]

Table S4. *B. gladioli* pv. *cocovenenans* and non-target strains were used to evaluate the specificity of LAMP and LAMP based visualization assay in this study.

Organism	No.	Bacterial species	Strain	Source	Genus-special target for LAMP
B. gladioli	1	*B. gladioli* pv. *cocovenenans*	CICC25108	a	+
	2	*B. gladioli* pv. *cocovenenans*	CICC25126	a	+
	3	*B. gladioli* pv. *cocovenenans*	CICC25131	a	+
	4	*B. gladioli* pv. *cocovenenans*	CICC25131	a	+
	5	*B. gladioli* pv. *cocovenenans*	BGC_0110	b	+
	6	*B. gladioli* pv. *cocovenenans*	BGC_0111	b	+
	7	*B. gladioli*	BG_0119	b	-
Non-target strains	8	*Burkholderia cepacia*	CICC10857	a	-
	9	*Burkholderia contaminans*	CICC23882	a	-
	10	*Burkholderia aenigmatica*	CICC24958	a	-
	11	*Burkholderia multivorans*	CICC25193	a	-
	12	*Burkholderia metalliresistens*	CICC10561	a	-
	13	*Burkholderia cenocepacia*	CICC25294	a	-
	14	*Pseudomonas aeruginosa*	ATCC 10104	c	-
	15	*Escherichia coli*	CMCC 44103	d	-
	16	*Salmonella enteritidis*	CMCC 50335	d	-
	17	*Bacillus cereus*	ATCC 14579	c	-
	18	*Listeria monocytogenes*	ATCC 19114	c	-
	19	*Staphylococcus aureus*	ATCC 29213	c	-
	20	*Campylobacterjejuni*	ATCC 6633	c	-

a: CICC, China Center of Industrial Culture Collection, China. b:Laboratory isolate. c: ATCC, American Type Culture Collection, USA. d: CMCC, China Medical Culture Collection, China.

+/-: positive and negative signals.

Table S5. Comparison of LAMP assays for the detection of *B. gladioli* pv. *cocovenenans*.

System name	Target	Amplification	Signal output material	Visual sensitivity	Detection time	Refs
Colorimetric-LAMP-based visual intelligent detection	bacteria	LAMP	fluorescence dye	6.68×10^2 CFU/25 g	70 min	[23]
LAMP-based lateral flow biosensor	*bon M*	LAMP	Fe_3O_4@PDA	1.0×10^4 CFU/mL	60 min	[24]
LAMP-based lateral flow dipstick technique	*Group_0 918*	LAMP	Au@PDA	4.1×10^3 CFU/g	60 min	Our study

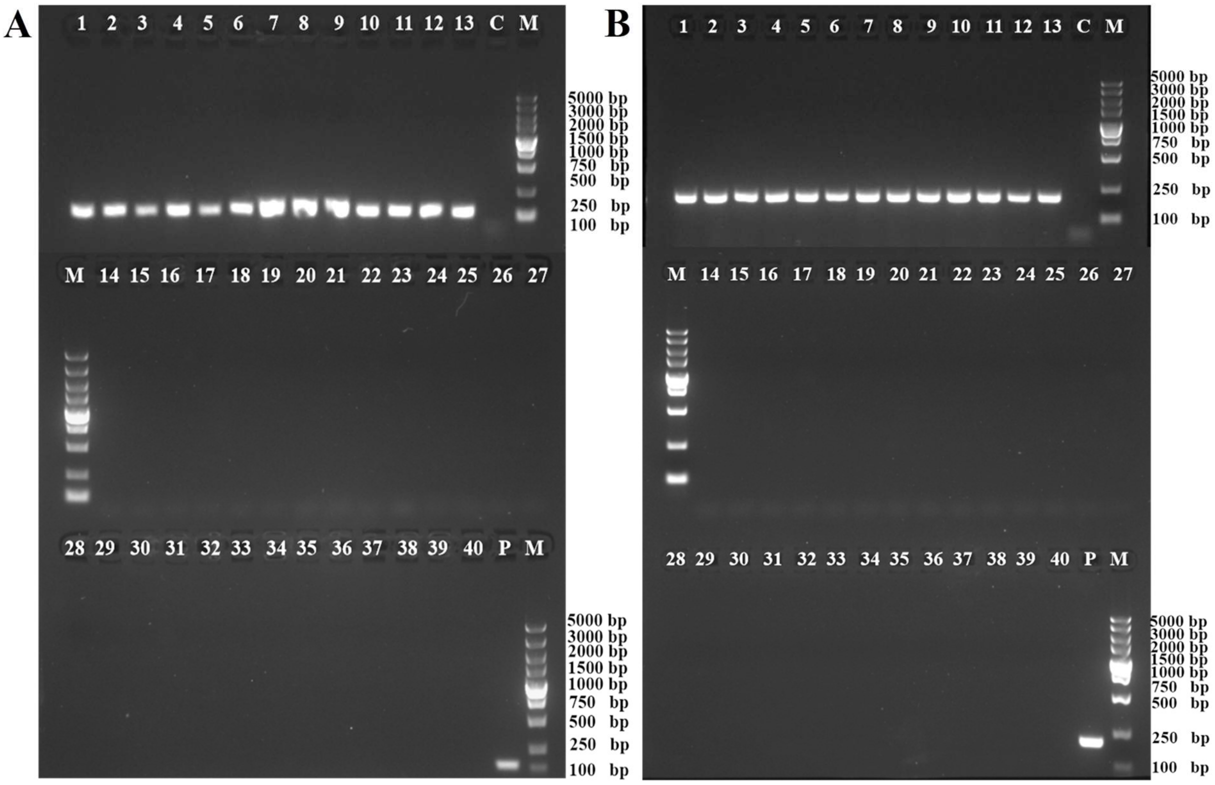

Figure S1. The specificity evaluation of novel targets for *B. gladioli* pv. *cocovenenans* by PCR assay. PCR results of novel molecular detection gene *group_0918* (A) and *group_0110* (B) specific for *B. gladioli* pv. *cocovenenans*; lanes 1-13: target *B. gladioli* pv. *cocovenenans* strains, lanes 14-40: other non-target *B. gladioli* pv. *cocovenenans* strains; all lane M: DL5000 DNA marker, P: positive control (genomic DNA of the *B. gladioli* pv. *cocovenenans* standard strain), C:negative control (nuclease-free water).

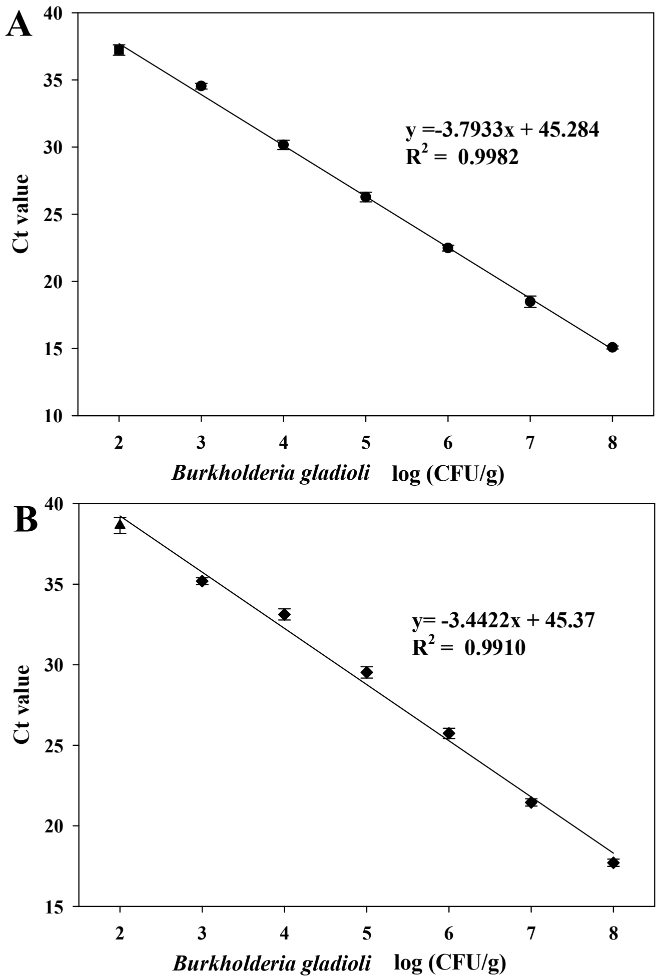

Figure S2. The qPCR results for detection of *B. gladioli* pv. *cocovenenans* in artificially contaminated sample. Establishment of standard curves for *group_0918* (A) and *group_0110* (B) based on the relationship between cycle threshold (Ct) values and the logarithmic values of the cell numbers of *B. gladioli* pv. *cocovenenans* in a range of 10^2 ~ 10^8 CFU/g. All results were collected in triplicate, with error bars representing the standard deviation of three independent replicates.

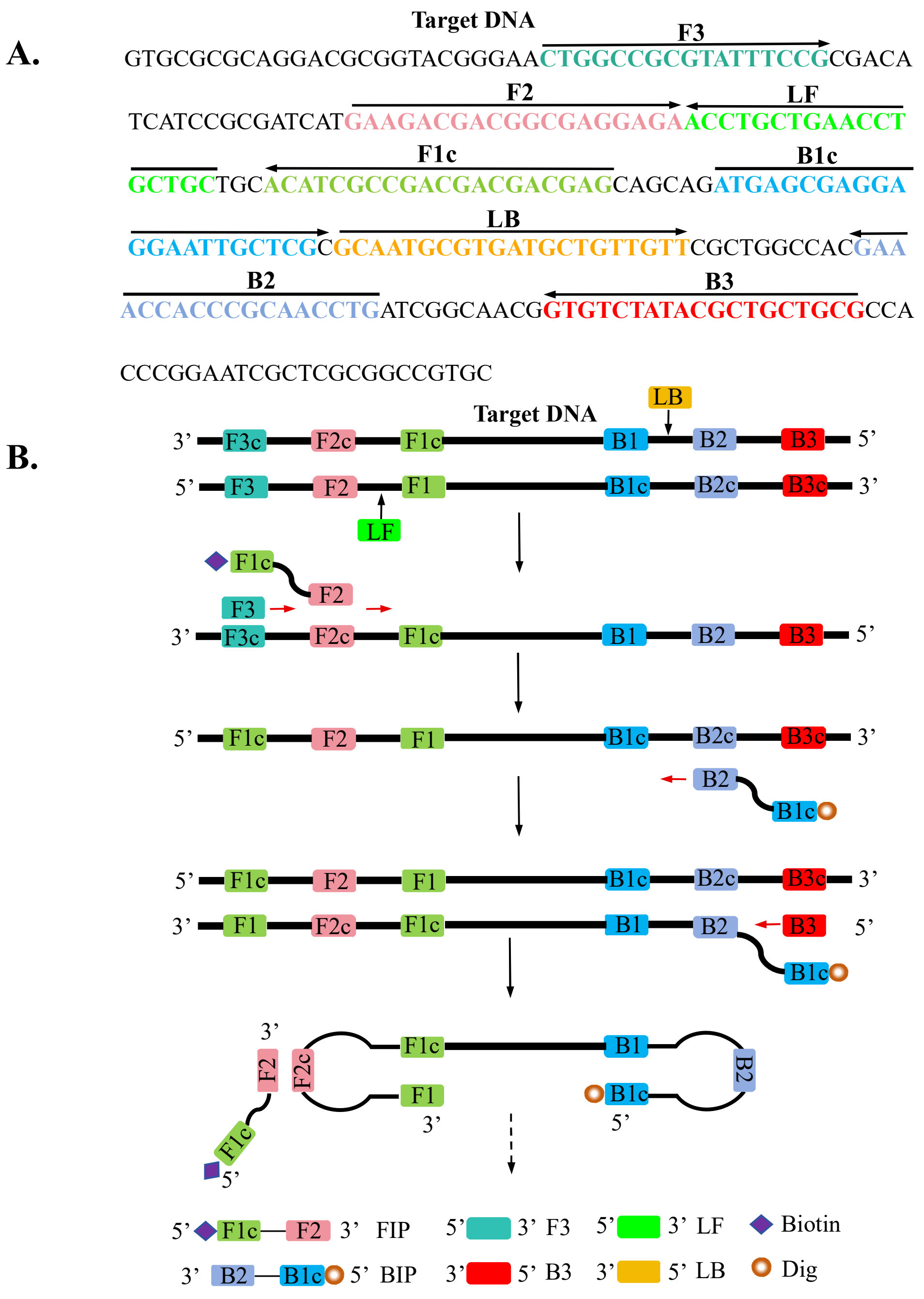

Figure S3. Schematic representation of molecular amplification of LAMP (localization of the LAMP modified primers on target DNA sequence (A) and major steps in the LAMP reaction(B)) .

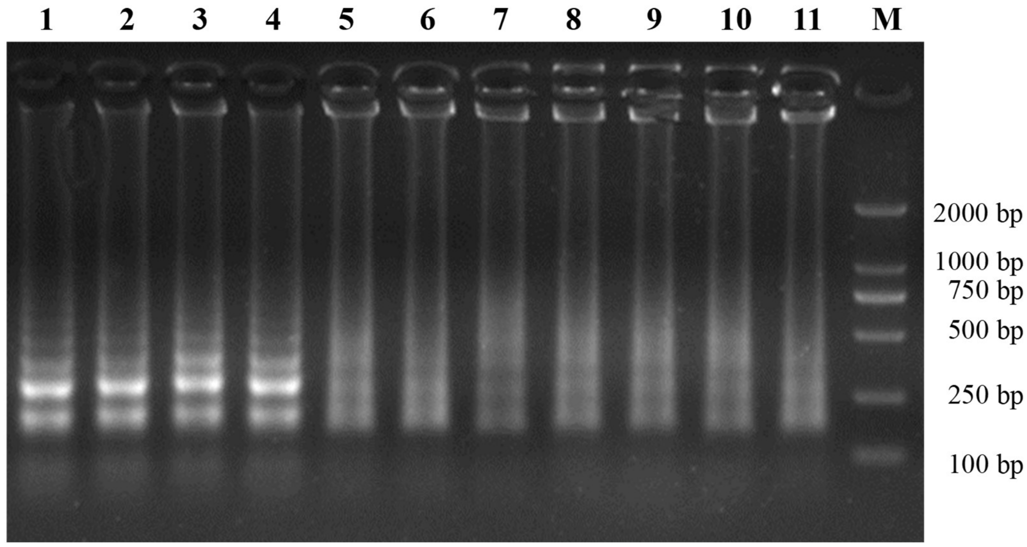

Figure S4. Optimization of the reaction temperature for LAMP assay.
M: DL2000 DNA marker, lanes 1-11: 68, 67, 66, 65, 64, 63, 62, 61, 60, 59, and 58° C, respectively.
